# Needling and open filtering bleb revision after XEN-45 implantation—a retrospective outcome comparison

**DOI:** 10.1007/s00417-021-05204-1

**Published:** 2021-05-11

**Authors:** Stefan Steiner, Hemma Resch, Barbara Kiss, Daniel Buda, Clemens Vass

**Affiliations:** Department of Ophthalmology and Optometry, Medical University of Vienna, Währinger Gürtel 18-20, 1090 Vienna, Austria

**Keywords:** XEN, Needling, Bleb revision, MIGS, Glaucoma surgery, Filtering surgery

## Abstract

**Purpose:**

To compare efficacy and safety of needling and open bleb revision after XEN-45 surgery.

**Methods:**

This retrospective study represents real-life data of patients who underwent XEN-45 surgery between November 2014 and June 2018 in the Vienna General Hospital. The following groups were formed for data evaluation: (PSEA) primary surgery secondary intervention allowed (*n* = 268); (PS) primary surgery until secondary intervention (*n* = 268); (N) first needling until additional secondary intervention (*n* = 55); (BR) first bleb revision until additional secondary intervention (*n* = 105). Main outcome measures were pre- and postoperative intraocular pressure (IOP), number of glaucoma medication (GM), Kaplan–Meier success rates, and secondary intervention rates. Success was defined as postoperative IOP < 21 mmHg and < 18 mmHg together with ≥ 20% IOP reduction with medication allowed.

**Results:**

IOP (and GM) was lowered from 23.5 ± 8.0 (GM 3.1 ± 1.0) to 14.9 ± 8.2 mmHg (1.2 ± 1.4) in group PSEA and 18.1 ± 8.2 mmHg (1.5 ± 1.4) in group PS, in group N from 23.2 ± 10.1 (1.5 ± 1.0) to 19.3 ± 8.5 mmHg (2.2 ± 1.3) and in group BR from 22.0 ± 8.0 mmHg (2.5 ± 1.1) to 15.5 ± 6.4 mmHg (1.3 ± 1.5) after a median follow-up of 16.0, 8.4, 4.8, and 7.3 months, respectively. Success rates at 1 year were significantly higher in group BR (50.7%) compared to PS (37.7%, *p* = 0.019) and N (24.3%; *p* = 0.015). An additional intervention was required less frequently in group BR (17.1%) compared to group PS (49.6%, *p* < 0.001) and group N (54.5%, *p* < 0.001).

**Conclusion:**

Our data appear to indicate favorable outcomes for open XEN bleb revision in terms of Kaplan–Meier success rates and secondary intervention rate compared to the needling procedure.


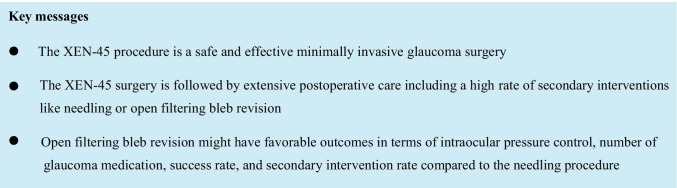


## Introduction

Trabeculectomy is the most commonly performed glaucoma surgery with a good intraocular pressure (IOP) lowering effect [1]. Nevertheless, many types of minimally invasive glaucoma surgery (MIGS) like iStent [2–4] (Glaukos Corporation, Laguna Hills, CA), trabectome [3, 5] (NeoMedix, Tustin, CA), canaloplasty [6], CyPass [7] (Alcon, Fort Worth, TX), Hydrus [4] (Ivantis, Inc, Irvine, CA), and Kahook dual blade [8] (New World Medical Inc., Rancho Cuamonga, CA) were introduced to provide an improved safety profile, similar IOP reduction compared to trabeculectomy, and less postoperative care and interventions. These new methods have in common that IOP regulation is not as good as with a traditional trabeculectomy even though the safety profile is preferable in MIGS [9].

The XEN-45 (Allergan Inc., Irvine, CA) implant by Allergan is a glaucoma drainage device aiming for well-controlled long-term IOP reduction and a good safety profile with uncomplicated postoperative patient management. Current literature shows that satisfying postoperative IOP levels can be reached but at the expense of very frequent bleb needlings or surgical revisions [10–16]. Despite some evidence on the effectiveness of needling [17] and open filtering bleb revision [18] after XEN-45, data about the XEN-45 surgery itself, without needling or revision, as well as comparative data about the effectiveness of bleb revision vs. needling are still missing.

## Materials and methods

In this retrospective study, we included all patients who underwent XEN-45 gel stent implantation at the Vienna General Hospital in the period from November 2014 to June 2018. No exclusion criteria were defined. The study was approved by the local ethics committee and followed the tenets of the Declaration of Helsinki.

The surgical technique of XEN-45 was the usual ab interno implantation following a preoperative subconjunctival injection of 0.05 to 0.1 ml of either 0.1 mg/ml or 0.2 mg/ml Mitomycin C (MMC) solution. Open bleb revision was performed in a similar way as published recently by Linton [18]. First, a six- to 9-mm wide conjunctival peritomy was performed. To have enough access, we usually supplemented this with a nasally located radial conjunctival incision. As soon as the implant was located, scar tissue was carefully removed around the external tip of the XEN-45. In the event that no flow was achieved, the device was intubated with a 10–0 or 9–0 nylon suture (*n* = 5), or if unsuccessful (*n* = 1), or in case of inadvertent damage to the XEN-45, the eye was given a new shunt (*n* = 15). Eyes that received a new shunt were also included in our analysis. In most cases, tenonectomy was performed in an area approximately one clock hour position to both sides of the XEN-45 and as far posterior as achievable. At the end of the procedure, the conjunctiva was closed with 8–0 vicryl sutures, and we subconjunctivally injected 0.1 ml of 0.2 mg/ml MMC solution.

Bleb needling was done at the slit lamp using a 27G needle or the bent needling knife (Kai Industries Co., Ltd., Oyana, Japan), followed by subconjunctival injection of 0.1 ml of 0.2 mg/ml MMC solution.

Main outcome measures were pre- and postoperative IOP, pre- and postoperative number of glaucoma medication (GM), complications (hypotony, reduction of anterior chamber depth, choroidal detachments, maculopathy, hyphema, infection), secondary interventions (defined as needling or open filtering bleb revision), and other secondary glaucoma surgery. Hypotony was defined as IOP < 6 mmHg at any single measurement and reduction of anterior chamber depth as peripheral iridocorneal touch or worse. Additionally, we report the Kaplan–Meier success rates for the different groups and success criteria.

**Fig. 1:**
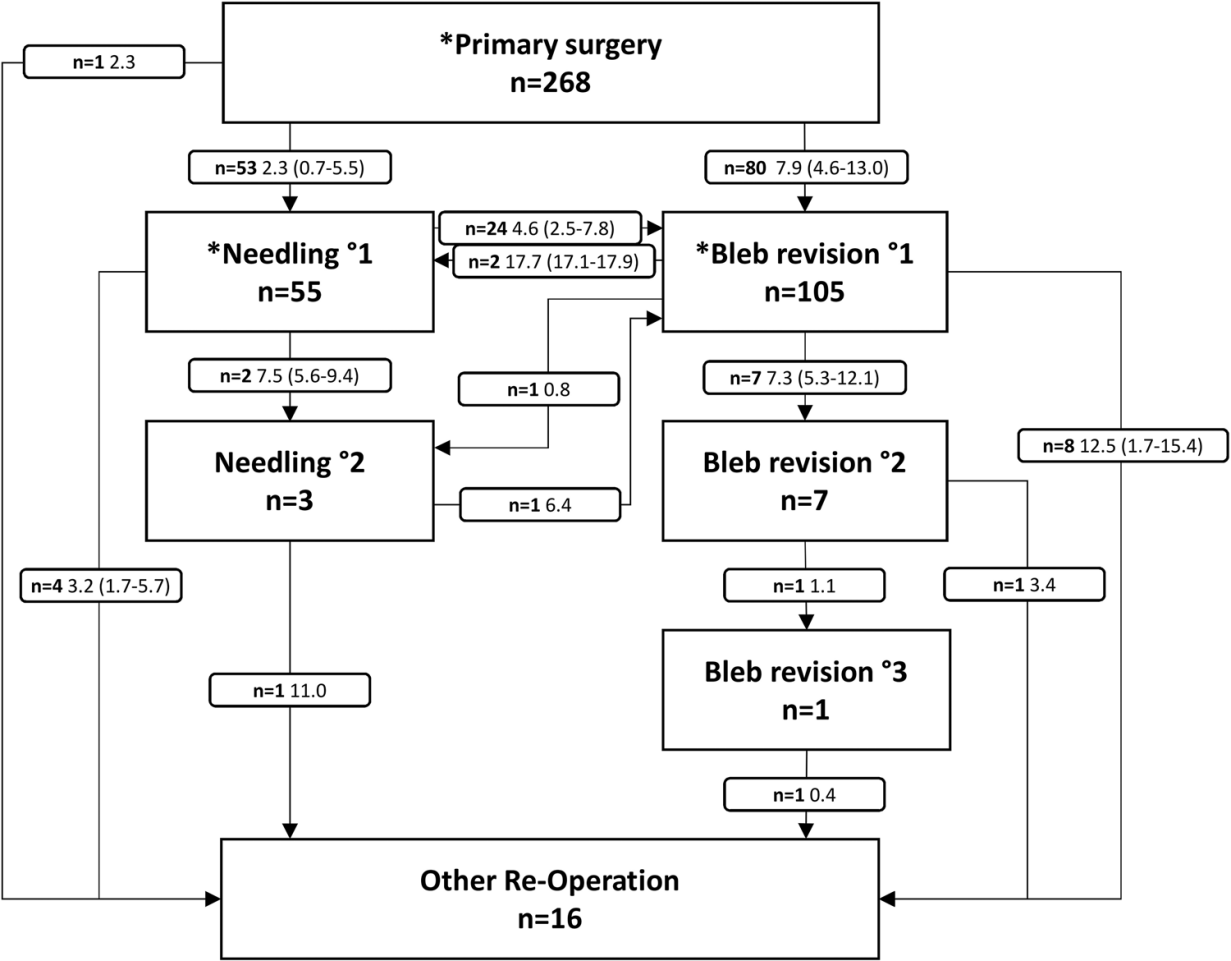
Additional interventions following Xen primary surgery; number of patients, median interval in months, in parenthesis = interquartile range; *included and analyzed groups

Four groups were formed for data evaluation: (1) primary surgery with everything allowed (PSEA); (2) primary surgery (PS); (3) needling (N); (4) bleb revision (BR). All eyes were included in the PSEA group, where any number of secondary interventions was allowed, but any other secondary glaucoma surgery was considered a failure. The PS group also comprised all eyes, but for this group as well as for the N and BR group, any secondary intervention or secondary glaucoma surgery was deemed a failure. Any first needling after primary surgery was included in the N group, and similarly, any first bleb revision was included in the BR group. Included groups are shown in Fig. 1.

Patient data were retrieved retrospectively from patient files and were categorized in the following time periods: day 1–2, day 3–7, day 8–14, day 15–30, month > 1–2, month > 2–6, month > 6–12, month > 12–24, month > 24–36, month > 36–48, and month > 48 until last follow-up. Up to three most recent preoperative data records that determined the surgical indication were considered for data analysis individually for the PSEA/PS, N, and the BR group.

Qualified **s**uccess was defined as IOP below 21 mmHg and below 18 mmHg together with an IOP reduction of 20% or more (QS21 and QS18) and glaucoma medication allowed. Another success criterion was 20% or more IOP reduction or reduction in the number of Glaucoma Medication of 2 or more (IoGM). This was done to reflect the praxis that in some patients, the main goal of the XEN-45 was to reduce glaucoma medication.

Kaplan–Meier survival analysis was carried out to compare failure rates of the PS, N, and BR group with the success criteria QS21, QS18, and IoGM. Failure was determined as not meeting the mentioned criteria on two consecutive visits after 60 days of follow-up or at the last visit.

Subgroup analysis was performed to evaluate success in eyes with a preoperative IOP of ≤ 21 mmHg and a preoperative IOP of > 21 mmHg and to analyze differences in success between stand-alone XEN-45 implantation and combined XEN-45 surgery with cataract extraction and intraocular lens implantation.

Statistical analyses were performed using SPSS® Version 21. Paired *t* test was used to compare pre- and postoperative IOP and GM changes. The unpaired *t* test was used to compare pre- and postoperative IOP and GM between our defined groups at certain time periods. Chi-square test was used to calculate differences in the occurrence of additional interventions and complications between groups. Success rates in our Kaplan–Meier analyses were compared using the Log Rank test pairwise over strata. Multivariate cox proportional hazards models were calculated to stratify baseline risk factors for failure in the PS group and the BR vs. N group. All tests were two-sided with *p* values < 0.05 considered as statistically significant.

## Results

The baseline characteristics of the study groups are shown in Table 1. A total of 268 eyes of 222 patients who underwent XEN-45 surgery were included in our analysis. The median age of the whole cohort was 68.2 (54.0–76.6) years. The ratio between right and left eyes was 142 to 126, and the male to female ratio was 130 to 138. The XEN-45 surgery was performed as a stand-alone procedure in 193 eyes and as combined procedure with cataract extraction in 75 eyes.

**Table 1:** Baseline characteristics

	Primary surgery	Needling	Bleb revision
	*n* = 268	*n* = 55	*n* = 105
Age (years), median (IQR)	68.2 (54.0–76.6)	67.8 (52.1–77.0)	68.48 (54.1–76.8)
Race, *n* (%)			
Caucasian	264 (99)	55 (100)	103 (98)
Asian	4 (1)	0 (0)	2 (2)
Gender, *n* (%)			
Female	130 (49)	27 (49)	52 (49)
Male	138 (51)	28 (51)	53 (51)
IOP (mm Hg) and GM, mean ± SD			
IOP baseline	23.5 ± 8.0	23.2 ± 10.1	22 ± 8.0
GM baseline	3.1 ± 1.0	1.5 ± 1.4	2.5 ± 1.1
Diagnosis, *n* (%)			
Primary open angle	79 (29)	16 (29)	30 (29)
Primary open angle (normal tension)	16 (6)	2 (4)	6 (6)
Pseudoexfoliative	60 (22)	13 (24)	22 (21)
Pigment dispersion	16 (6)	4 (7)	7 (7)
Primary angle closure	29 (11)	6 (11)	12 (11)
Juvenile open angle	21 (8)	2 (4)	6 (6)
Secondary glaucoma*	22 (8)	5 (9)	8 (8)
Ocular hypertension	20 (7)	6 (11)	10 (10)
Congenital glaucoma	5 (2)	1 (2)	4 (4)
Previous eye surgery or laser, *n* (%)			
Posterior intraocular lens	83 (31)	13 (24)	34 (32)
Trabeculectomy	27 (10)	6 (11)	10 (10)
Trabeculotomy	7 (3)	1 (2)	4 (4)
Baerveldt	3 (1)	1 (2)	1 (1)
Ahmed	2 (1)	0 (0)	1 (1)
iStent	4 (1)	0 (0)	1 (1)
Hydrus	1 (0)	1 (2)	0 (0)
Kahook	2 (1)	0 (0)	0 (1)
Cyclodestructive procedure	5 (2)	2 (4)	3 (3)
Laser peripheral iridotomy	26 (10)	8 (15)	10 (10)
Laser trabeculoplasty	10 (4)	2 (4)	5 (5)
Laser iridoplasty	5 (2)	0 (0)	3 (3)

*IQR* interquartile range

^*^Secondary glaucoma excluding pseudoexfoliative and pigment dispersion glaucoma

**Fig. 2:**
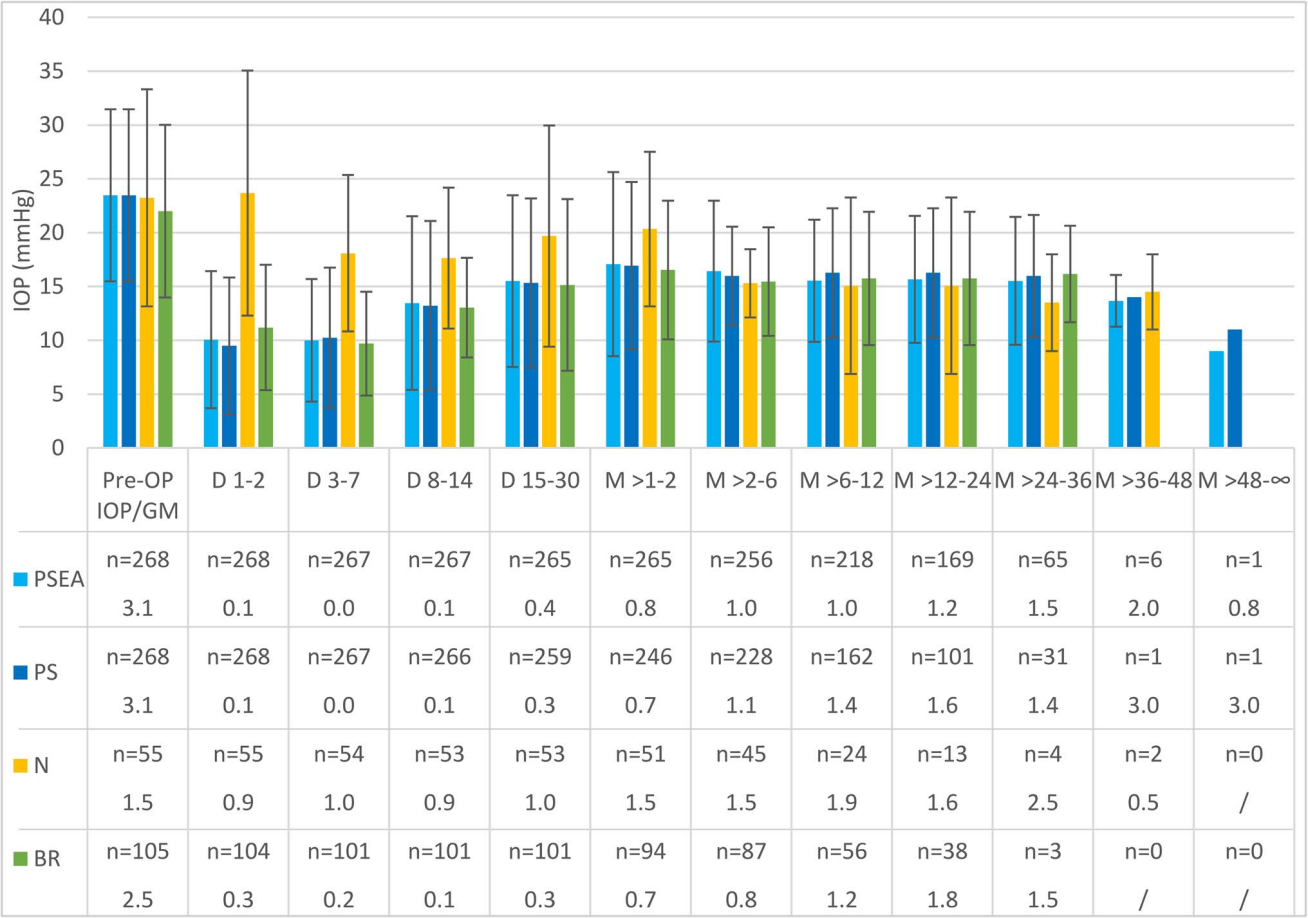
IOP and glaucoma medication over time in the groups PSEA (primary surgery everything allowed), PS (primary surgery), N (needling), BR (bleb revision), D (day), and M (month)

Baseline IOP in group PSEA/PS was 23.5 mmHg and was lowered to 14.9 mmHg in the PSEA group and to 18.1 mmHg in the PS group after a median follow-up of 16.0 vs. 8.4 months, respectively. The number of GM was reduced from 3.1 to 1.2 in group PSEA and 1.5 in group PS. In group N and group BR, the preoperative IOP was 23.2 vs. 22.0 mmHg and was lowered to 19.3 vs. 15.5 mmHg. GM changed from 1.5 vs. 2.5 to 2.2 vs. 1.3 after a median follow-up period of 4.8 vs. 7.3 months in the N vs. BR group, respectively. The time course of IOP and GM is shown in Fig. 2. No significant differences in baseline IOP were observed between group PS, N, and BR. Baseline GM was significantly higher in group PS compared to group N (*p* < 0.001) and BR (*p* < 0.001). GM at baseline was also higher in group BR than in group N (*p* < 0.001). From the immediate postoperative period throughout the first 2 years, IOP and GM were very similar between PSEA, PS, and BR. Contrary to this, the N group had significantly higher IOP during the first 2 months, followed by a reduction to the same level compared to all other groups after the second month.

Following XEN-45 implantation, in 53 eyes, the first secondary intervention was needling, whereas it was bleb revision in 80 eyes. While 25 eyes (47%) of the former needed subsequent bleb revision, only two eyes (2.5%) of the latter required subsequent needling. These eyes, receiving their first needling after previous bleb revision or vice versa, were also included in either the N group or BR group, which made a total of 55 eyes in the N group and 105 eyes in the BR group.

All secondary interventions after XEN-45 primary surgery with median intervals are shown in the flow chart in Fig. 1. Three out of 55 eyes in the N group needed a second needling, 25 eyes a bleb revision, and five eyes required any other glaucoma surgery, whereas in the BR group, seven out of 105 eyes needed a second and one eye a third bleb revision, three eyes required needling, and eight eyes any other glaucoma surgery. In total, other glaucoma surgeries were necessary in 16 eyes (ten trabeculectomies, one re-trabeculectomy, three Ahmed valve implants, one Baerveldt implant, and one cyclodestructive procedure). The cyclodestructive procedure was performed after XEN-45 primary surgery without any needling or bleb revision in a patient after retinal detachment surgery. An additional IOP lowering secondary intervention or other secondary glaucoma surgery was required less frequently in Group BR (*n* = 18/105, 17.1%) compared to group PS (*n* = 133/268, 49.6%, *p* < 0.001) and group N (*n* = 30/55, 54.5%, *p* < 0.001), while no significant difference was observed between group PS and N.

### Success rates

**Fig. 3:**
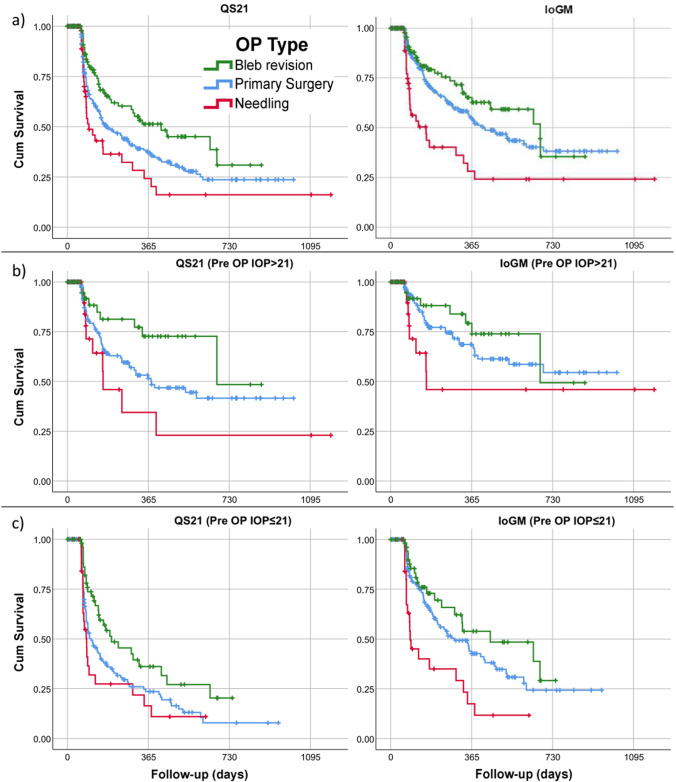
Kaplan–Meier plots comparing success rates of groups PS (primary surgery), N (needling), and BR (bleb revision) according to our success criteria (**a**) in the whole cohort, (**b**) in the subgroup with a preoperative IOP of > 21 mmHg, and (**c**) in the subgroup with a preoperative IOP of ≤ 21 mmHg, IoGM (20% or more IOP reduction or reduction in the number of glaucoma medication of 2 or more)

Kaplan–Meier curves of the PS, N, and BR group for the QS21 and IoGM criteria are shown in Fig. 3a. Kaplan–Meier success rates (and standard error) for the QS21 criteria at 1 year were 37.7% (3.4%), 24.3% (7.8%), and 50.7% (6.1%) for the PS, N, and BR group, respectively. For the stricter QS18 criteria, the success rates were lower, and for the IoGM criteria, the success rates were higher compared to the QS21 criteria. For both the QS21 and the QS18 criteria, survival was significantly better in the BR group compared to the PS (log rank, QS21 *p* = 0.019; QS18 *p* = 0.016) and N group (log rank QS21 *p* = 0.015; QS18 *p* = 0.016), while there was no difference between the PS and N group. Survival for the IoGM criteria was lower in group N compared to BR (log rank *p* = 0.003) and PS (log rank *p* = 0.039) and there was no difference between group PS and BR.

The Kaplan–Meier curves of the subgroups with a preoperative IOP of > 21 mmHg are shown in Fig. 3b and with preoperative IOP of ≤ 21 mmHg in Fig. 3c. Patients with a preoperative IOP of > 21 mmHg showed better survival rates at one year in the PS group (log rank QS21 *p* < 0.001; QS18 *p* < 0.001; IoGM *p* < 0.001) and in the BR group (log rank QS21 *p* = 0.002; QS18 *p* < 0.001, IoGM n.s.) compared to the subgroup with IOP ≤ 21 mmHg. No differences were observed in the N group for the QS21, QS18, and IoGM criteria. Regarding the subgroup with IOP ≤ 21 mmHg, the success criterion IoGM yielded better survival compared to the QS21 or the QS18 criteria. The latter two were virtually identical. Again, the survival was better for the BR group compared to both other groups.

The combined XEN-45 primary surgery with cataract extraction showed a tendency towards better Kaplan–Meier survival rates compared to the stand-alone procedure in the QS21 and QS18 criteria. Nevertheless, this difference was not statistically significant. Also, no significant differences were observed between eyes with prior stand-alone and combined primary surgeries in the N and BR group for the QS21, QS18, and IoGM criteria (Table 2).

**Table 2:** Median survival time and standard error (SE) in days for the primary surgery (PS), needling (N), and bleb revision (BR) group and for the subgroups stand-alone vs. combined primary XEN-45 implantation with cataract extraction using the QS21, QS18, and IoGM success criteria*

	Overall		Stand-alone		Combined	
	Median survival		Median survival		Median survival	
	Estimate	SE	Estimate	SE	Estimate	SE
Primary surgery	*n* = 268		*n* = 193		*n* = 75	
QS21	176	31	155	14	278	47
QS18	151	21	148	9	247	47
IoGM	420	63	405	72	425	105
Needling	*n* = 55		*n* = 38		*n* = 17	
QS21	97	22	87	18	125	50
QS18	87	8	86	5	97	31
IoGM	159	45	114	54	161	119
Bleb revision	*n* = 105		*n* = 82		*n* = 23	
QS21	423	134	423	156	449	93
QS18	323	101	338	138	323	62
IoGM	673	158	673	18	449	116

SE, standard error

^*^QS21 and QS18, postoperative IOP < 21 mmHg and < 18 mmHg together with ≥ 20% IOP reduction; IoGM, 20% or more IOP reduction or reduction in the number of glaucoma medication of 2 or more

Hazard ratios calculated with the use of Cox proportional hazards models are shown in Table 3. In the model of the PS group, higher baseline IOP (HR = 0.94 per mmHg, *p* < 0.001) and a combined XEN-45 surgery with cataract extraction (HR = 0.55, *p* = 0.01) lowered the risk of failure. All other covariates were not statistically significant. No interactions between type of glaucoma and combined or stand-alone procedures were observed in a sub-model.

**Table 3:** Hazard ratios for not meeting the QS21 criterium within the primary surgery (PS) group and needling (N) vs. bleb revision (BR) groups*

	Primary surgery		Needlings and bleb revisions	
Covariates	Hazard ratio (95% CI)	*P* value	Hazard ratio (95% CI)	*P* value
Sex: male	0.85 (0.60–1.20)	0.35	1.99 (1.19–3.35)	0.01
Age	1.00 (0.99–1.01)	0.95	1.00 (0.97–1.02)	0.66
IOP baseline	0.94 (0.91–0.97)	< 0.001	0.89 (0.85–0.94)	< 0.001
GM baseline	0.94 (0.78–1.15)	0.56	0.99 (0.78–1.24)	0.90
Diagnosis: primary open angle ^1^	1.00	0.50	1.00	0.62
Diagnosis: PEX or pigmentary ^1^	0.72 (0.47–1.11)	0.13	0.84 (0.46–1.55)	0.59
Diagnosis: angle closure ^1^	0.99 (0.55–1.78)	0.96	0.59 (0.24–1.44)	0.25
Diagnosis: other ^1^	0.91 (0.51–1.64)	0.76	0.65 (0.28–1.50)	0.31
Combined XEN with cataract extraction	0.55 (0.36–0.85)	0.01		
Conjunctival opening surgery prior to XEN	0.82 (0.56–1.20)	0.31		
Revision type: bleb revision			0.38 (0.21–0.70)	< 0.001
Needling prior to bleb revision			1.20 (0.55–2.59)	0.65
Bleb revision prior to needling			2.35 (0.40–13.95)	0.35
Interval to primary surgery			1.00 (1.00–1.00)	0.75

^*^Hazard ratios calculated using Cox proportional hazards models

^1^Primary open angle was defined as the reference diagnosis

In the N vs. BR group model, BR as revision type was strongly associated with a lower risk of failure regarding the QS21 criteria (HR = 0.38, *p* < 0.001). Higher baseline IOP was a protective factor (HR = 0.89 per mmHg, *p* < 0.001). Male gender was a risk factor for failure (HR = 1.99, *p* = 0.02). All other putative factors, including the interval to primary surgery, were not statistically significant.

### Complications

All three procedures, the XEN-45 implantation, the needling, and the bleb revision were followed by a small number of complications (Table 4). Transient ocular hypotony occurred in 30.2% after PS, 17.1% after BR, and 5.5% after N with statistically significant differences (*p* < 0.05) between all groups. The average duration of ocular hypotony was 12 days after PS, 7 days after N, and 7 days after BR. The time of hypotony exceeded 30 days in 4 patients of the PS group and lasted 30 or fewer days in all cases of the N and BR group. There was no case with persistent ocular hypotony. Shallow anterior chamber was significantly more frequent in PS (4.1%) than in N (0%) and BR (0%). Choroidal detachments or folds were significantly more frequently observed after PS (14.2%) compared to N (0.0%) and BR (0.0%). The average duration of choroidal detachments or folds in the PS group was 28 days, whereas 5 choroidals lasted longer than 30 days. Kissing choroidal detachments or choroidals with macular involvement occurred in 5 eyes after PS only. All patients who suffered choroidal detachments received atropine eye drops (1% or 2%) once daily until complete spontaneous recovery, while sometimes this was supported by anterior chamber reformation (*n* = 5). One kissing choroidal detachment originated from a delayed choroidal hemorrhage which was surgically drained. In 35.4% of the patients after PS, at least one of the mentioned complications occurred. For the N and BR group, that holds true for 7.2% and 22.9% of the patients. These differences were statistically significant between all groups (PS vs. N *p* < 0.001, PS vs. BR *p* = 0.019, N vs. BR *p* = 0.033).

**Table 4:** Complications after primary surgery (PS), needling (N), and bleb revision (BR)

	Primary surgery	Needling	Bleb revision
	*n* = 268	*n* = 55	*n* = 105
Complications, *n* (%)			
Transient hypotony	81 (30)***	3 (6)	18 (17)
Shallow anterior chamber ^§^	11 (4)*	0 (0)	0 (0)
Choroidal detachment/folds	38 (14)**	0 (0)	0 (0)
Bleb leak	5 (2)	1 (2)	4 (4)
Corneal dellen	4 (2)	0 (0)	2 (2)
Any complication per eye	95 (35)***	4 (7)	24 (23)

^§^Shallow anterior chamber defined as peripheral iridocorneal touch or worse; (p < 0.05 comparing * PS vs. BR, ** PS vs. BR and N, *** difference between all groups)

## Discussion

IOP reduction after XEN-45 implantation in our cohort is comparable with other studies where IOP was lowered from 20.0–25.1 mmHg at baseline to 13.1–17.1 mmHg at 12 months [10–16]. Long-term XEN-45 efficacy data exceeding 12 months of follow-up are still limited. Data at 24 months are available from Gabbay et al. [13] and Reitsamer et al. [16] where they reported an IOP of 14.5–15.2 at 0.5–1.1 GM, which is well consistent with our IOP at that timepoint albeit with a higher number of GM in our data.

The needling or bleb revision rate (49.6%) was high in our cohort. Depending on different treatment strategies, previous studies reported either more needlings or bleb revisions. While Widder et al. [14] followed a strategy to perform only bleb revisions (revision rate 34.0% at 8.5 months mean follow-up) and no needlings, the group of Gabbay et al. [13] rather relied on needlings (Needling rate 36.8% at 24 months). In general, the rate for secondary interventions was between 22.1 and 44.0% in the studies mentioned above [10–16].

Midha et al. [17] conducted a prospective trial to evaluate needling outcomes in 51 eyes, where IOP was lowered from 23.6 to 14.3 mmHg at an average follow-up time of 17.0 months. As no medication values were given and multiple needlings were allowed, a comparison to our results appears difficult. However, following a needling procedure, in Midhas case series, about 30% of the cases required reoperation, whereas in our cohort, 53% required either open bleb revision (45%) or a secondary glaucoma surgery (8%). The discrepancy in these numbers may be explained by the different approaches towards patients with failed needling. While they performed a second needling in about 40%, we did so in only 4% and instead relied on the effect of an open bleb revision.

Linton et al. [18] analyzed outcomes of a XEN-45 revision surgery method, similar to our bleb revision strategy, including 16 eyes resulting in an IOP reduction from 26.1 mmHg with 2.0 GM to 16.3 mmHg with 0.7 GM after 1 year of follow-up. A further needling after bleb revision was required in four and a further drainage surgery in three of 16 eyes. In our case series, we performed bleb revisions in 105 eyes, out of which one required bleb needling, seven required a second bleb revision, and ten eyes required secondary glaucoma surgery. Linton et al. report a QS21 of 56% at 1 year, which is well comparable to our QS21 of 50.7%. It is noteworthy that we reported the crude success of bleb revision not allowing for any further intervention, while in Linton’s series, two out of the 9 eyes counted as success at 12 months postoperatively even though they required intermittent bleb needling. Subtracting these two eyes, their QS21 would have been 44% instead of 56%.

To the best of our knowledge, this is the first study to present real-life data of bleb needling (N) and open bleb revision (BR) after XEN-45 implantation where no further intervention was allowed to get comparable results of the specific procedure. Furthermore, we report results of a group of patients allowing for postoperative needling and bleb revision (PSEA) as part of the planned procedure and compare this to the results of pure XEN-45 surgery (PS), not allowing for any further intervention. Kaplan–Meier survival analyses resulted in survival rates being markedly different between the groups. At 1 year postoperatively for the QS21 and the QS18 criteria, the survival was significantly better for the BR group compared to the PS and the N group while the latter two were not significantly different from each other. The IoGM criterion (IOP reduction or medication reduction) resulted in a lower success rate in the N group compared to the PS and the BR group while there was no difference between PS and BR group. Together, these findings suggest that in our study cohort, open bleb revision is superior to slit-lamp-based bleb needling and even better than the primary surgery itself. This finding is also supported by the median survival times, which were significantly longer for the BR (QS21 = 423 days) compared to the PS group (QS21 = 176 days).

The more favorable results of open bleb revision compared to primary XEN-45 implantation are surprising. Usually, in glaucoma surgery, a surgical revision would not be expected to surpass the primary surgery in terms of efficacy. Due to its retrospective nature, our study cannot be taken as definite proof of the superiority of BR over PS. For further conclusions about this, we should await other confirming evidence. However, our results might be explained by the inherently uncertain position of the external end of the XEN-45 relative to the conjunctiva or Tenon’s capsule. This circumstance may trigger a scarring response in some cases which is avoided after open bleb revision because of a clear position of the XEN-45 below the conjunctiva and—if not resected—the Tenon’s capsule. Another possible reason for our findings might be the fact that we usually resected the cicatrized Tenon’s capsule in the approximately two clock hour positions adjacent to the XEN-45.

No differences in success were observed between stand-alone XEN-45 implantation and combined XEN-45 surgery with cataract extraction, although the Cox proportional hazards model revealed a lower hazard ratio for failure in the combined group (HR = 0.55, *p* = 0.01). We think that a combination of factors may have caused this finding: a larger proportion of angle-closure glaucoma in the combined cases (24.0 vs. 5.7%), lower preoperative IOP in the combined cases (20.7 ± 6.3 vs. 24.5 ± 8.6 mmHg), higher failure risk for eyes with lower preoperative IOP, but still comparable success rates.

Since success rates for the subgroup with a preoperative IOP < 21 mmHg showed more unsatisfactory results in the QS21, QS18, and IoGM criteria, our data suggest that XEN-45 is a less suitable option, especially in patients with low preoperative IOP values.

Due to the retrospective nature of the study and the open design, we cannot exclude an effect of selection and detection bias. Our cohort was characterized by a large attrition rate where good cases may have been discharged while bad cases remained in our out-patient clinic for regular checkups. Furthermore, our treatment strategy led to IOP values reflecting the preferences of the treating physicians by masking not acceptable IOP values with constant needlings and bleb revisions. As we started to implant the XEN-45, needling was the preferred revision strategy. Later, we got the impression that bleb revision leads to preferable results. This resulted in 24% of the bleb revisions being preceded by needlings, while only 4% of the needlings were preceded by a bleb revision. For a more precise analysis, a prospective approach with longer follow-up and sophisticated methods is needed.

## Conclusion

The XEN-45 implant is an effective and safe MIGS device. However, in our study cohort, 49.6% of eyes needed at least one postoperative secondary intervention. Although previous studies present data on needling and bleb revision outcomes after XEN-45 implantation, no comparison between these two postoperative interventions has been reported. In the present study, our data appear to indicate that open filtering bleb revision might have beneficial outcomes in terms of Kaplan–Meier success rates, and the reintervention rate compared to the needling procedure.

## Data Availability

On demand.

## References

[1] Kirwan JF, Lockwood AJ, Shah P, Macleod A, Broadway DC, King AJ (2013). Trabeculectomy in the 21st century: a multicenter analysis. Ophthalmology.

[2] Arriola-Villalobos P, Martínez-de-la-Casa JM, Díaz-Valle D, Fernández-Pérez C, García-ánchez J, García-Feijoó J (2012). Combined iStent trabecular micro-bypass stent implantation and phacoemulsification for coexistent open-angle glaucoma and cataract: a long-term study. Br J Ophthalmol.

[3] Gonnermann J, Bertelmann E, Pahlitzsch M, Maier-Wenzel AKB, Torun N, Klamann MKJ (2017). Contralateral eye comparison study in MICS & MIGS: Trabectome® vs. iStent inject®. Graefe’s Arch Clin Exp Ophthalmol.

[4] Ahmed IIK, Fea A, Au L, Ang RE, Harasymowycz P, Jampel HD (2020). A prospective randomized trial comparing Hydrus and iStentmicroinvasive glaucoma surgery implants for standalone treatment of open-angle glaucoma: the COMPARE study. Ophthalmology.

[5] Kaplowitz K, Bussel II, Honkanen R, Schuman JS, Loewen NA (2016). Review and meta-analysis of ab-interno trabeculectomy outcomes. Br J Ophthalmol.

[6] Lin ZJ, Xu S, Huang SY, Bin ZX, Zhong YS (2016). Comparison of canaloplasty and trabeculectomy for open angle glaucoma: a meta-analysis. Int J Ophthalmol.

[7] Reiss G, Clifford B, Vold S, He J, Hamilton C, Dickerson J (2019). Safety and effectiveness of CyPasssupraciliary micro-stent in primary open-angle glaucoma: 5-year results from the COMPASS XT study. Am J Ophthalmol..

[8] Salinas L, Chaudhary A, Berdahl JP, Lazcano-Gomez GS, Williamson BK, Dorairaj SK (2018). Goniotomy using the Kahook Dual Blade in severe and refractory glaucoma: 6-month outcomes. J Glaucoma.

[9] Lavia C, Dallorto L, Maule M, Ceccarelli M, Fea AM (2017) Minimally-invasive glaucoma surgeries (MIGS) for open angle glaucoma: a systematic review and meta-analysis. PLoS ONE 12:1–33 10.1371/journal.pone.0183142 PMC5574616 28850575

[10] Mansouri K, Gillmann K, Rao HL, Guidotti J, Mermoud A (2018). Prospective evaluation of XEN gel implant in eyes with pseudoexfoliative glaucoma. J Glaucoma.

[11] Gillmann K, Bravetti GE, Mermoud A, Rao HL, Mansouri K (2019). XEN gel stent in pseudoexfoliative glaucoma: 2-year results of a prospective evaluation. J Glaucoma.

[12] Heidinger A, Schwab C, Lindner E, Riedl R, Mossböck G (2019). A retrospective study of 199 Xen45 stent implantations from 2014 to 2016. J Glaucoma.

[13] Gabbay IE, Allen F, Morley C, Pearsall T, Bowes OM, Ruben S (2019) Efficacy and safety data for the XEN45 implant at 2 years: a retrospective analysis. Br J Ophthalmol 104(8):1125–1130 10.1136/bjophthalmol-2019-313870 31727624

[14] Widder RA, Dietlein TS, Dinslage S, Kühnrich P, Rennings C, Rössler G (2018). The XEN45 Gel Stent as a minimally invasive procedure in glaucoma surgery: success rates, risk profile, and rates of re-surgery after 261 surgeries. Graefe’s Arch Clin Exp Ophthalmol.

[15] Smith M, Charles R, Abdel-Hay A, Shah B, Byles D, Lim LA (2019). 1-year outcomes of the Xen45 glaucoma implant. Eye.

[16] Reitsamer H, Sng C, Vera V, Lenzhofer M, Barton K, Stalmans I (2019). Two-year results of a multicenter study of the ab interno gelatin implant in medically uncontrolled primary open-angle glaucoma. Graefe’s Arch Clin Exp Ophthalmol.

[17] Midha N, Gillmann K, Chaudhary A, Mermoud A, Mansouri K (2020). Efficacy of needling revision after XEN gel stent implantation: a prospective study. J Glaucoma.

[18] Linton E, Au L (2020) Technique of Xen implant revision surgery and the surgical outcomes: a retrospective interventional case series. Ophthalmol Ther 9(1):149–157 10.1007/s40123-020-00234-0 PMC7054468 32062789

